# Association of ADIPOQ gene variants with body weight, type 2 diabetes and serum adiponectin concentrations: the Finnish Diabetes Prevention Study

**DOI:** 10.1186/1471-2350-12-5

**Published:** 2011-01-10

**Authors:** Niina Siitonen, Leena Pulkkinen, Jaana Lindström, Marjukka Kolehmainen, Johan G Eriksson, Mika Venojärvi, Pirjo Ilanne-Parikka, Sirkka Keinänen-Kiukaanniemi, Jaakko Tuomilehto, Matti Uusitupa

**Affiliations:** 1 Department of Clinical Nutrition and Food and Health Research Centre, Institute of Public Health and Clinical Nutrition, University of Eastern Finland, Kuopio, Finland; 2 Department of Health Promotion and Chronic Disease Prevention, National Institute for Health and Welfare, Helsinki, Finland; 3 Department of Public Health, University of Helsinki, Helsinki, Finland; 4 Unit of General Practice, Helsinki University Central Hospital, Helsinki, Finland; 5 Folkhalsan Research Centre, Helsinki, Finland; 6 Department of General Practice and Primary Health Care, University of Helsinki, Helsinki, Finland; 7 Vasa Central Hospital, Vasa, Finland; 8 Population Studies Unit, Department of Chronic Disease Prevention, National Institute for Health and Welfare, Turku, Finland; 9 Diabetes Centre, Finnish Diabetes Association, Tampere, Finland; 10 Science Centre, Pirkanmaa Hospital District, Tampere University Hospital, Tampere, Finland; 11 Institute of Health Sciences, University of Oulu, Oulu, Finland; 12 Unit of General Practice, Oulu University Hospital, Oulu, Finland; 13 South Ostrobothnia Central Hospital, Seinäjoki, Finland; 14 Research Unit, Kuopio University Hospital, Kuopio, Finland

## Abstract

**Background:**

Adiponectin, secreted mainly by mature adipocytes, is a protein with insulin-sensitising and anti-atherogenic effects. Human adiponectin is encoded by the *ADIPOQ *gene on the chromosomal locus 3q27. Variations in *ADIPOQ *are associated with obesity, type 2 diabetes (T2DM) and related phenotypes in several populations. Our aim was to study the association of the *ADIPOQ *variations with body weight, serum adiponectin concentrations and conversion to T2DM in overweight subjects with impaired glucose tolerance. Moreover, we investigated whether *ADIPOQ *gene variants modify the effect of lifestyle changes on these traits.

**Methods:**

Participants in the Finnish Diabetes Prevention Study were randomly assigned to a lifestyle intervention group or a control group. Those whose DNA was available (n = 507) were genotyped for ten *ADIPOQ *single nucleotide polymorphisms (SNPs). Associations between SNPs and baseline body weight and serum adiponectin concentrations were analysed using the univariate analysis of variance. The 4-year longitudinal weight data were analysed using linear mixed models analysis and the change in serum adiponectin from baseline to year four was analysed using Kruskal-Wallis test. In addition, the association of SNPs with the risk of developing T2DM during the follow-up of 0-11 (mean 6.34) years was analysed by Cox regression analysis.

**Results:**

rs266729, rs16861205, rs1501299, rs3821799 and rs6773957 associated significantly (p < 0.05) with body weight at baseline and in the longitudinal analyses. The rs266729 C allele and the rare minor alleles of rs2241766 and rs2082940 were associated with an increased adjusted hazard ratio of developing T2DM. The differences in baseline serum adiponectin concentrations were seen according to rs16861210, rs17366568, rs2241766, rs6773957 and rs2082940 and differences in the change of serum adiponectin levels from baseline to the four year examination were seen according to rs16861205, especially in subjects who were able to lose weight during the first year of intervention.

**Conclusions:**

These results from the Finnish Diabetes Prevention Study support the concept that genetic variation in *ADIPOQ *locus contributes to variation in body size and serum adiponectin concentrations and may also modify the risk of developing T2DM.

**Trial registration number:**

ClinicalTrials.gov NCT00518167

## Background

Type 2 diabetes (T2DM) and obesity are increasing worldwide. Although this is mainly due to environmental factors, such as changes in diet and lifestyle, much evidence for genetic predisposition to these complex traits exist [1].

Adiponectin is an adipokine, and its plasma levels are decreased in obesity [2], T2DM [3], insulin resistance [4], dyslipidemia [5], and coronary artery disease (CAD) [6]. In humans, weight reduction increases serum adiponectin levels [3,7], and in mice, chronic administration of recombinant adiponectin leads to enhanced fatty acid oxidation, and weight loss [8] with beneficial effects on lipid metabolism and insulin sensitivity [9]. Furthermore, adiponectin knock-out mice are highly sensitive to diet induced insulin resistance [10].

Adiponectin is encoded by the *ADIPOQ *gene on chromosome 3q27, a region identified as susceptibility locus for the metabolic syndrome and T2DM by genome wide scans [11-13]. In various study populations, *ADIPOQ *SNPs and haplotypes associate with phenotypes related to obesity [14-18], insulin resistance and T2DM [14-16,19-27] and serum adiponectin levels [14,16,18,21,23,28-34]. However, possibly due to differences in metabolic or ethnic backgrounds of the participants in different studies, the results of previous genetic association studies are conflicting. In addition, most of the studies performed so far have been cross-sectional and have only included few SNPs in the region of *ADIPOQ*. The aim of the present study was to provide supportive evidence for the involvement of *ADIPOQ *variation in T2DM and obesity related phenotypes and serum adiponectin levels in the prospective Finnish Diabetes Prevention Study (DPS). Moreover, the aim was to examine whether *ADIPOQ *SNPs modify the effect of lifestyle changes on these traits.

## Methods

### Subjects and study design

The DPS is a randomised, controlled multicentre study with five participating clinics in Finland. The study design has been described in detail earlier [35,36]. The main inclusion criteria were BMI > 25 kg/m^2^, age 40-64 years, and impaired glucose tolerance (IGT) based on the mean value of two consecutive oral glucose tolerance tests (OGTTs). The diagnosis of IGT and T2DM were based on WHO 1985 criteria [37]. IGT was defined as fasting plasma glucose < 7.8 mmol/l and a 2-h plasma glucose 7.8-11.0 mmol/l (OGTT, glucose load 75 g). A total of 522 subjects (BMI 31.1 ± 4.6 kg/m^2^) were randomly allocated into one of the two groups: an intensive diet and exercise intervention group or a control group. Randomisation was stratified according to the clinic attended, sex and 2-hour plasma glucose concentration. The study protocol was approved by Ethics Committee of the National Public Health Institute in Helsinki, Finland and a written informed consent was received from all subjects [35,36]. We certify that all applicable institutional and governmental regulations concerning the ethical use of human volunteers were followed during this research.

### Intervention

The subjects in the intervention group received individualized counselling on diet and exercise [38]. The five goals of the intervention were: ≥ 5% or more reduction in body weight, reduction in the intake of total fat to ≤ 30% and of saturated fat to ≤ 10% of daily energy intake, increase of the intake of dietary fibre to at least 15 g per 1000 kcal and moderate-to-vigorous exercise at least 30 min per day. The subjects in the control group received general information about healthy diet and exercise at baseline.

### Examinations

A medical history was recorded and a physical examination with anthropometric measurements was performed at baseline and at each annual follow-up visit. Measurements recorded at baseline and 1, 2, 3 and 4 year examinations were used in the present study.

### Assessment of glucose and insulin metabolism

A 2-h OGTT was performed at baseline and annually. Plasma glucose and insulin samples were drawn at 0 min and 120 min during the OGTT with a glucose load of 75 g. Plasma glucose was measured locally by standard methods as previously described [36]. Serum insulin was measured by RIA (Phadaseph Insulin RIA 100, Pharmacia Diagnostica, Uppsala, Sweden). The intra-assay coefficient of variation was 5.3% and the interassay coefficient of variation was 7.6%. The diagnosis of IGT was based on the mean value of two oral glucose tolerance tests. A new diagnosis of diabetes (fasting plasma glucose ≥7.8 mmol/l or 2-h plasma glucose ≥11.0 mmol/l) was confirmed by a second oral glucose tolerance test.

### Determination of adiponectin concentrations

Fasting serum adiponectin levels were measured using an enzyme-linked immunosorbent assay (ELISA) (B-Bridge International, Inc., San Jose, CA, USA), on whole plasma stored at -80°C. The intra-assay and inter-assay coefficients of variation were 5.5-7.9% and 6.5%, respectively. Frozen serum samples for adiponectin measurements were only available from subset of participants from three study clinics (n = 243 at baseline, and n = 209 at 4-year examination). In altogether 190 subjects both baseline and year four serum adiponectin concentrations were measured.

### Selection of SNPs and genotyping

DNA sample was available from 507 subjects (166 men and 341 women). Tagging SNPs with minimum minor allele frequency of 5% were selected based on genotype data of the Hapmap CEU population [39] by using the Tagger algorithm [40] with rs2241766 and rs1501299, selected based on previous literature, being forced into the selection. Altogether, the ten SNPs selected for genotyping covered 73% of the variation within *ADIPOQ *region (20 kbp) with r^2 ^≥ 0.8. Rs2241766 and rs1501299 were genotyped using PCR followed by SNaPshot ddNTP Primer Extension Kit technique (ABI Prism; Applied Biosystems) as described earlier [24]. Rs17366568 was genotyped using PCR-RFLP method. The genomic DNA was amplified by PCR by using the following primers: forward 5'-CCCAATAGTCAAACATGTGC-3' and reverse 5'-TCATCCTTGGAAGACCAACC-3' followed by digestion with restriction enzyme MseI (New England Biolabs). Other SNPs were genotyped with TaqMan Allelic Discrimination assays according to manufacturer's instructions by using the ABI PRISM 7000 sequence detector (Applied Biosystems, Foster City, CA). For a subset of randomly selected samples (6.3%) genotyping was repeated in order to calculate success rate.

### Statistical analysis

The data were analysed with SPSS for Windows 14.0 (SPSS Inc, Chicago, IL). Chi square test was used to test the departure of genotypes from Hardy-Weinberg equilibrium (HWE) and the distribution of genotypes between study groups. Skewed variables were transformed for analysis and are reported as back-transformed geometric means and 95% confidence intervals (CIs), or were analysed with nonparametric tests. Normally distributed data are presented as mean ± SE. All analyses were carried out using three inheritance models: additive, dominant (common allele homozygotes compared with heterozygotes and minor allele homozygotes), and recessive (common allele homozygotes and heterozygotes compared with minor allele homozygotes). The baseline differences in continuous variables between genotypes were evaluated with the univariate ANOVA, general linear model (GLM). Longitudinal weight data were examined using the linear mixed models analysis. Normality was assessed by plotting the residuals. Association of the SNPs with conversion to T2DM was analysed by using Cox regression. Adjustments for age, sex, BMI, waist-to-hip ratio (WHR), fasting plasma glucose and study group (intervention or control) were made when appropriate. A multiple SNP analysis was performed for groups of SNPs that associated with baseline weight, baseline serum adiponectin levels and the risk of T2DM by including all significant SNPs (coded as major allele homozygotes vs. minor allele carriers) in the same statistical model and then removing SNPs sequentially starting from the least significant one. Sex-genotype or study group-genotype interaction term was included in analyses when appropriate. When significant interaction (p < 0.05) was observed, men and women or the study groups were analysed separately. Change in serum adiponectin concentration was calculated by subtracting the baseline values from the fourth year values. Correction for multiple hypothesis testing for single SNP analyses was performed with false discovery rate (FDR) method using Q value 1.0 software [41]. The q-values were calculated separately for each trait, but all SNPs and inheritance models were included in the same calculations. The pair wise linkage disequilibrium (LD) between individual SNPs was evaluated with Haploview software (version 3.32; Broad institute, Cambridge, MA) [42]. Haploblocks were defined using the default algorithm [43].

## Results

Table 1 shows the clinical and metabolic characteristics of the study population.

**Table 1:** Baseline characteristics, 1-year weight change and 4-year serum adiponectin change in DPS participants.

Sex (male/female)		166/341
Age (y)		55.33 ± 7.06 (507)
Weight (kg)		86.20 ± 14.19 (507)
BMI (kg/m^2^)		31.25 ± 4.54 (507)
Waist circumference (cm)		101.22 ± 10.99 (505)
Fasting plasma glucose (mmol/l)		6.14 ± 0.75 (507)
2-h plasma glucose (mmol/l)		8.88 ± 1.49 (507)
Fasting serum insulin (mU/l)		14.77 ± 7.40 (461)
2-h serum insulin (mU/l)		95.17 ± 65.10 (458)
Serum adiponectin (μg/ml)		8.47 ± 3.74 (237)
1-year weight change (kg)		-2.736 ± 4.723 (506)
	Intervention group	-4.465 ± 4.976 (256)
	Control group	-0.965 ± 3.695 (250)
4-year adiponectin change (μg/ml)		0.015 ± 2.135 (190)
	Intervention group	0.091 ± 2.245 (97)
	Control group	-0.063 ± 2.203 (93)

Values are mean ± SD (N); BMI, Body Mass Index

### Genotype frequencies and linkage disequilibrium (LD) patterns

**Figure 1:**
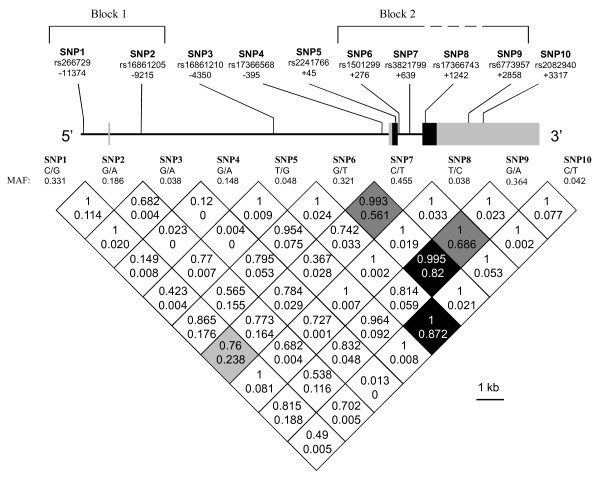
***ADIPOQ *gene, locations of the genotyped variants and their pairwise linkage disequilibrium (LD) patterns**. Schematic presentation of the *ADIPOQ*, indicating the locations of the analysed variants, the two observed haploblocks and the pairwise LD measures D' (above) and r^2 ^(below). Grey boxes, untranslated region; black boxes, coding region; MAF, minor allele frequency.

Ten SNPs were genotyped from the 507 DNA samples of the DPS participants with an error rate of 0% and a call rate of 100% in replicated samples for all markers. The locations of the analysed variants are presented in Figure 1 with pairwise LD measures (D' and r^2^). As previously reported, we observed two LD blocks [31] with rs266729 and rs16861205 constituting the block 1, and rs1501299, rs3821799 and rs6773957 constituting the block 2, while other SNPs remained between or outside the blocks.

Genotype counts and allele frequencies are shown in Table 2. All SNPs, except rs16861210, were in HWE (p < 0.05) in the entire DPS population. The deviation from HWE could be explained as a chance finding due to low frequency of the minor allele and limited sample size of DPS. Thus, rs16861210 was included in further analyses. Genotype frequencies of rs16861205, rs3821799 and rs2082940 differed significantly (p < 0.05) between the intervention and control group of DPS. Again, this is probably due to chance and these SNPs were included in analyses, but all longitudinal data analyses were performed separately in both study groups in addition to analyses in the total study population.

**Table 2:** *ADIPOQ *genotype counts and allele frequencies.

	Genotypes (count)			Allele freq (%)		p/q^a^	p/q^b^
rs266729	CC	CG	GG	C	G		
	219	240	48	66.9	33.1	0.124/0.683	0.509/0.300

rs16861205	GG	GA	AA	G	A		
	337	151	19	81.4	18.6	0.685/0.855	0.005/0.138

rs169861210	GG	GA	AA	G	A		
	471	33	3	96.2	3.8	0.007/0.683	0.279/0.195

rs17366568	GG	GA	AA	G	A		
	371	122	14	85.2	14.8	0.306/0.683	0.876/0.412

rs2241766	TT	TG	GG	T	G		
	460	45	2	95.2	4.8	0.431/0.683	0.107/0.138

rs1501299	GG	GT	TT	G	T		
	234	220	53	67.9	32.1	0.903/0.855	0.134/0.138

rs3821799	CC	CT	TT	C	T		
	149	255	103	54.5	45.5	0.748/0.855	0.017/0.138

rs17366743	TT	TC		T	C		
	468	39		96.2	3.8	0.368/0.683	0.758/0.396

rs6773957	GG	GA	AA	G	A		
	206	233	68	63.6	36.4	0.869/0.855	0.071/0.138

rs2082940	CC	CT	TT	C	T		
	466	39	2	95.8	4.2	0.234/0.683	0.047/0.138

^a^p/q value for Hardy-Weinberg equilibrium

^b^p/q value for differences in genotype frequencies between the study groups of DPS

Correction for multiple hypothesis testing was performed with FDR, denoted as q-value.

### Genotype associations with body weight

Rs266729, rs16861205, rs1501299, rs3821799 and rs6773957 associated with baseline body weight, when adjusted for age and sex (table 3). Furthermore, the same SNPs associated with 4-year follow-up measurements of weight, when analysed longitudinally with adjustments for age, sex, study group and the time variable. Similar associations between *ADIPOQ *SNPs and baseline and 4-year measurements of BMI were seen (data not shown). Genotype differences were mainly seen by using additive and dominant inheritance models.

**Table 3:** Significant associations between *ADIPOQ *SNPs and baseline body weight

				p^a^/q	p^b^/q
rs266729 (N)	CC (219)^c^	CG, GG (288)^d^			
All	85.04 (83.33-86.79)	87.66 (86.09-89.25)		0.025/0.024	0.020/0.700
male	91.36 (88.60-94.21)	89.69 (87.26-92.19)		0.385/0.420	
female	80.18 (78.16-82.25)	84.46 (82.63-86.33)		0.003/0.036	

rs16861205 (N)	GG (337)	GA (151)	AA (19)		
All	87.66 (86.20-89.14)	84.37 (82.33-86.46)	83.86 (78.36-89.67)	0.023/0.024	0.528/0.684
	GG (337)	AA, AG (170)			
All	87.65 (86.20-89.13)	84.31 (82.39-86.26)		0.006/0.024	0.302/0.684

rs1501299 (N)	GG (234)	GT (220)	TT (53)		
All	88.09 (86.37-89.84)	85.36 (83.63-87.13)	84.16 (80.80-87.66)	0.032/0.024	0.486/0.684
	GG (234)	GT, TT (273)			
All	88.09 (86.37-89.84)	85.13 (83.56-86.72)		0.011/0.024	0.407/0.684

rs3821799 (N)	CC (149)	CT (255)	TT (103)		
All	89.63 (87.49-91.82)	85.64 (84.02-87.29)	83.99 (81.56-86.48)	0.001/0.024	0.187/0.700
	CC (149)	CT, TT (358)			
All	89.63 (87.49-91.82)	85.16 (83.78-86.57)		0.0005/0.024	0.072/0.724
	CC, CT (404)	TT (103)			
All	87.13 (85.80-88.48)	84.03 (81.59-86.55)		0.029/0.024	0.861/0.684

rs6773957 (N)	GG (206)	GA (233)	AA (68)		
All	88.31 (86.49-90.17)	85.29 (83.61-87.01)	84.98 (81.95-88.12)	0.033/0.024	0.200/0.700
	GG (206)	AA, AG (301)			
All	88.31 (86.50-90.17)	85.22 (83.72-86.76)		0.009/0.024	0.172/0.684

Data are expressed as retransformed geometric mean (95% CI)

Correction for multiple hypothesis testing was performed with FDR, denoted as q-value.

^a ^adjusted for age and sex

^b ^p for sex-genotype interaction

^c ^Men: N = 74, Women N = 145

^d ^Men: N = 92, Women N = 196

Individuals carrying the rs266729 G allele had higher baseline body weight than those homozygous for C allele. A sex-genotype interaction was found for weight and significant differences were observed in women. Likewise, during the 4-year follow-up significant differences in weight according to rs266729 were only observed in women (p = 0.007/q = 0.038 for the dominant inheritance model and p = 0.027/q = 0.309 for sex-genotype interaction).

**Figure 2:**
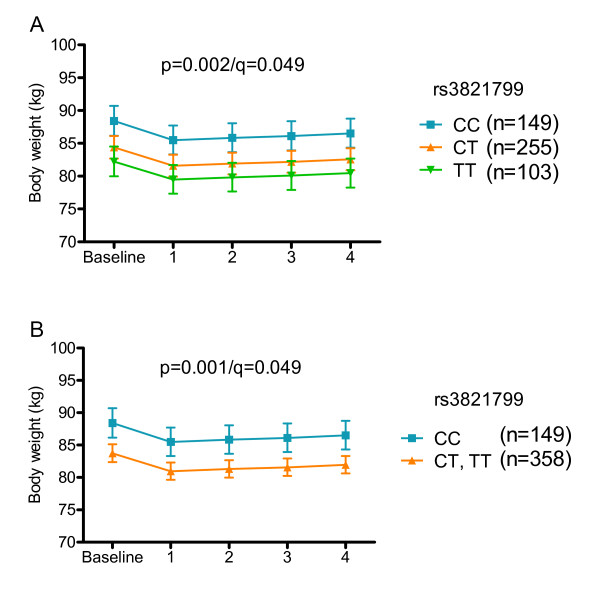
**Body weight from baseline to year 4 according to rs3821799**. Data are predicted geometric means and 95% CI. Additive (A) and dominant (B) inheritace models; p values are adjusted for age, sex, study group and the time; Correction for multiple hypothesis testing was performed with FDR, denoted as q-value.

Rs16861205 G, rs1501299 G, rs3821799 C and rs6773957 G alleles associated dose-dependently with higher baseline body weight. The longitudinal data is shown for rs3821799 in figure 2. Genotype differences remained significant during the 4-year follow-up, when the additive inheritance model was used (rs16861205: p = 0.028/q = 0.049, rs1501299:p = 0.041/q = 0.049, rs3821799: p = 0.002/q = 0.049, and rs6773957: p = 0.045/q = 0.049) and were even stronger when the dominant inheritance model was used (rs16861205: p = 0.008/q = 0.049, rs1501299: p = 0.014/0.049, rs3821799: p = 0.001/q = 0.049, and rs6773957: p = 0.014/q = 0.049). For rs3821799, the genotype differences were significant also when the recessive inheritance model was used. When the longitudinal analyses were performed separately in the intervention and control group, genotype differences remained constant during the follow-up in both groups, but were statistically significant only in the control group.

We also conducted multiple SNP models analysis with SNPs that associated individually with baseline body weight. In a model including all five SNPs, a trend for significance was observed for rs3821799 (p = 0.063). When the least significant SNPs were removed sequentially from the model, two SNPs remained in the final model: rs3821799 (p = 0.007) and rs16861205 (p = 0.101).

### Genotype associations with Type 2 Diabetes Incidence

Altogether 184 of 507 subjects converted from IGT to T2DM during the follow-up of 0-11 (mean 6.34) years. The association of the 10 *ADIPOQ *SNPs with the conversion from IGT to T2DM was analysed by using Cox regression models adjusting for baseline BMI, WHR, baseline fasting plasma glucose and study group (Table 4).

**Table 4:** Hazard ratios for association of *ADIPOQ *SNP1, rs2241766 and rs2082940 with T2DM

SNP	Genotype		Entire DPS		Intervention group		Control group
		N	HR (95% CI), p^a^/q	N	HR (95% CI), p^b^/q	N	HR (95% CI), p^b^/q
rs266729	CC	219	1	117	1	102	1
	CG	240	0.695 (0.513-0.942), 0.019/0.203	121	0.733 (0.453-1.185), 0.205/0.616	119	0.661 (0. 444-0.985), 0.042/0.237
	GG	48	0.615 (0.350-1.081), 0.091/0.330	21	0.400 (0.123-1.300), 0.128/0.616	27	0.743 (0.388-1.422), 0.370/0.818
	CC	219	1	117	1	102	1
	CG, GG	288	0.682 (0.510-0.912), 0.010/0.203	142	0.683 (0.428-1.090), 0.110/0.616	146	0.676 (0.464-0.985), 0.041/0.237

rs2241766	TT	460	1	231	1	229	1
	TG	45	1.521 (0.944-2.452), 0.085/0.330	28	1.320 (0.625-2.786), 0.466/0.616	17	1.648 (0.881-3.083), 0.118/0.470
	GG	2	5.448 (1.329-22.333), 0.019/0.203	0	-	2	4.993 (1.211-20.598), 0.026/0.237
	TT	460	1	231	1	229	1
	TG, GG	47	1.634 (1.035-2.579), 0.035/0.216	28	1.320 (0.625-2.786), 0.466/0.616	19	1.839 (1.028-3.289), 0.040/0.237

rs2082940	CC	466	1	233	1	233	1
	CT	39	1.383 (0.812-2.357), 0.233/0.668	26	1.356 (0.642-2.862), 0.425/0.616	13	1.352 (0.627-1.917), 0.442/0.821
	TT	2	5.368 (1.310-21.997), 0.020/0.203	0	-	2	4.885 (1.185-20.138), 0.028/0.237
	CC	466	1	233	1	233	1
	CT, TT	41	1.518 (0.918-2.512), 0.104/0.334	26	1.356 (0.642-2.862), 0.425/0.616	15	1.611 (0.813-3.196), 0.172/0.544

^a^Adjusted for baseline BMI, WHR, fasting plasma glucose and study group

^b^Adjusted for baseline BMI, WHR and fasting plasma glucose

Correction for multiple hypothesis testing was performed with FDR, denoted as q-value.

T2DM was less likely developed in study participants carrying rs266729 G allele. The adjusted hazard ratio was 0.682 (95% CI 0.510-0.912, p = 0.010/q = 0.203) for the G allele carriers compared with subjects with CC genotype, 0.695 (95% CI 0.513-0.942, p = 0.019/q = 0.203) for CG genotype and 0.615 (95% CI 0.350-1.081, p = 0.091/q = 0.330) for GG genotype. In addition, subjects carrying the rare minor alleles of rs2241766 and rs2082940 (G and T, respectively) had increased risk of developing T2DM. The results were similar when a statistical model not adjusted for BMI or WHR was used (data not shown).

Since the lifestyle intervention decreased the risk of T2DM significantly in the DPS [36], the effects of each SNP were examined for the intervention and control groups separately. The genotype differences were similar in both groups according to rs266729, but statistical significance was reached only in the control group. Only two subjects were homozygous for the rare alleles of rs2241766 and rs2082940 and both were in the control group. When dominant inheritance model was used, significant differences were only seen in the control group according to rs2241766.

In a multiple SNP model with rs266729, rs2241766 and rs2082940, only rs2766729 remained significant predictor of conversion from IGT to T2DM (adjusted hazard ratio 0.693, 95% CI 0.518-0.928, p = 0.014 for the G allele carriers compared with subjects with CC genotype). A borderline significance was observed for rs2241766 when it was included in the model with rs266729 (p = 0.086).

### Genotype associations with serum adiponectin levels

Baseline serum adiponectin levels differed significantly according to five *ADIPOQ *SNPs, when adjustments were made for age, sex and baseline WHR (Table 5): rs16861210 (p = 0.029/q = 0.032 for the additive and p = 0.008/q = 0.032 for the dominant inheritance model), rs17366568 (p = 0.003/q = 0.032 for the additive and p = 0.0007/q = 0.032 for the dominant inheritance model), rs2241766 (p = 0.056/q = 0.032 for TT vs. TG genotype), rs6773957 (p = 0.016/q = 0.032 for the additive, p = 0.021/q = 0.032 for the dominant and p = 0.022/q = 0.032 for the recessive inheritance model) and rs2082940 (p = 0.056/q = 0.032 for CC vs. CT genotype). Lower serum adiponectin concentrations were associated with rs16861210 G allele, rs17366568 A allele, rs2241766 TT genotype, rs6773957 G allele and rs2082940 CC genotype. In addition, a significant sex-genotype interaction was found for rs2241766 and rs2082940 (p = 0.020/q = 0.279 for both). When men and women were analysed separately, a significant association between rs2241766 and rs2082940 genotype and serum adiponectin levels was seen only in men (p = 0.003/q = 0.131 for both SNPs).

**Table 5:** Association of rs16861210, rs17366568, rs2241766, rs6773957 and s2082940 with baseline serum adiponectin levels

				p/q^a^	p/q^b^
rs16861210	GG (224)	GA (12)	AA (1)		
All	7.76 (7.32-8.22)	10.27 (8.29-12.45)	11.79 (5.33-20.77)	0.029/0.032	0.143/0.495
	GG (224)	AA, AG (13)			
All	7.76 (7.32-8.22)	10.38 (8.47-12.48)		0.008/0.032	0.201/0.495

rs17366568	GG (169)	GA (59)	AA (9)		
All	8.40 (7.88-8.94)	6.90 (6.16-7.69)	6.44 (6.48-8.48)	0.003/0.032	0.893/0.840
	GG (169)	AA, AG (68)			
All	8.40 (7.88-8.49)	6.84 (6.15-7.58)		0.0007/0.032	0.850/0.840

rs2241766	TT (224)	TG (13)			
All	7.82 (7.38-8.28)	9.70 (7.83-11.76)		0.056/0.032	0.020/0.279
Men^c^	6.45 (5.85-7.08)	12.54 (8.49-17.37)		0.003/0.131	
Women^d^	8.87 (8.32-9.44)	9.38 (7.30-11.71)		0.660/0.092	

rs6773957	GG (104)	AG (110)	AA (23)		
All	7.36 (6.74-8.00)	8.12 (7.51-8.75)	9.45 (8.05-10.96)	0.016/0.032	0.711/0.840
	GG (104)	AA, AG (133)			
All	7.35 (6.73-8.00)	8.34 (7.77-8.92)		0.021/0.032	0.559/0.840
	AG, GG (214)	AA (23)			
All	7.76 (7.31-8.22)	9.47 (8.06-10.99)		0.022/0.032	0.772/0.840

rs2082940	CC (224)	CT (13)			
All	7.82 (7.38-8.28)	9.70 (7.83-11.76)		0.056/0.032	0.020/0.279
Men^e^	6.45 (5.85-7.08)	12.54 (8.49-17.37)		0.003/0.131	
Women^f^	8.87 (8.32-9.44)	9.38 (7.30-11.71)		0.660/0.092	

Data are expressed as predicted geometric mean (95% CI)- Correction for multiple hypothesis testing was performed with FDR, denoted as q-value.

^a ^adjusted for age, sex and baseline WHR

^b ^sex-genotype interaction

^c^TT: N = 80, TG: N = 3; ^d^TT: N = 144, TG: N = 10; ^e^CC: N = 80, CT: N = 3; ^f^CC: N = 144, CT: N = 10

In multiple SNP model including rs16861210, rs17366568, rs6773957 and either rs2241766 or rs2082940, the first two SNPs remained significantly associated with baseline adiponectin levels (p = 0.012 and p = 0.003, respectively).

### Baseline serum adiponectin concentrations and T2DM risk

A trend for a lower T2DM risk was seen with hazard ratio of 0.695 (0.451-1.072, p = 0.100) for those with higher than median (6.250 for men and 8.815 for women) serum adiponectin concentrations compared with those with adiponectin lower than median.

### Genotype associations with 4-year change in adiponectin concentrations

**Figure 3:**
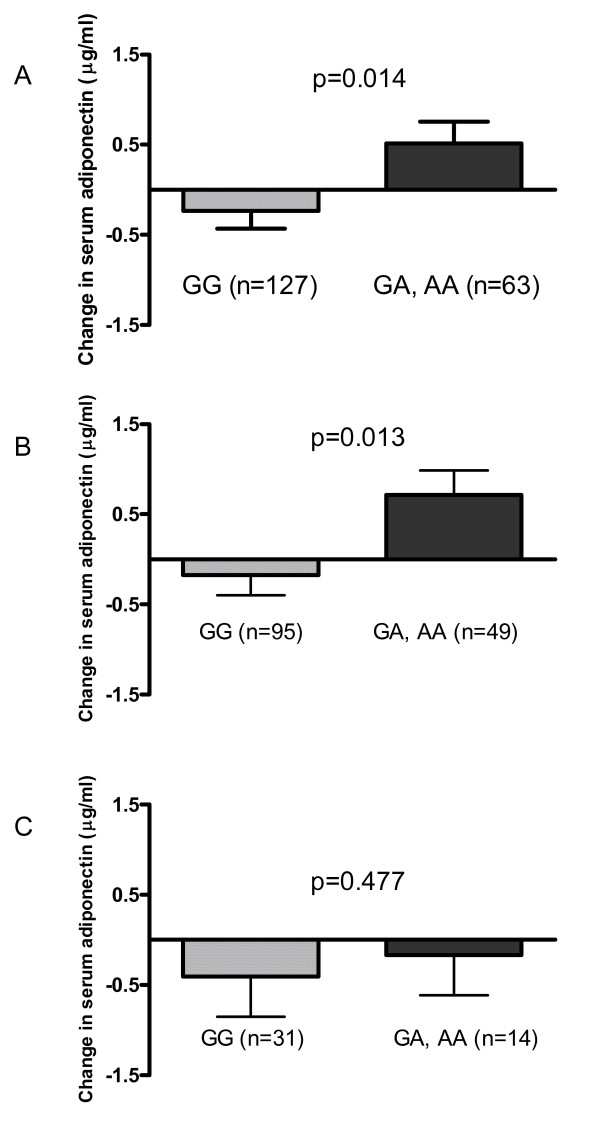
**Four year change in serum adiponectin levels according to rs16861205**. Data are means ± SD. Dominant inheritance model for the whole DPS (A), for participants who lost weight (B), and for participants who gained weight during the first year (C). p/q value for GG genotype vs. A allele carriers, Kruskal-Wallis test; Correction for multiple hypothesis testing was performed with FDR, denoted as q-value.

Rs16861205 associated with 4-year change in serum adiponectin concentrations (p = 0.040/q = 0.798 and p = 0.014/q = 0.798 for the additive and the dominant inheritance models, respectively) (figure 3). The A allele was dose dependently associated with increase in serum adiponectin levels (AA: n = 9 and AG: n = 54) compared with the GG genotype (n = 127). When study groups were analysed separately, genotype differences were similar, but statistical significance was not reached. When individuals who lost weight (n = 144) or gained weight (n = 45) during the first year of the intervention were analysed as separate groups, statistically significant differences in the 4-year change were seen only in those who were able to lose weight (p = 0.034/q = 0.725 for the additive inheritance model, p = 0.013/q = 0.725 for the dominant inheritance model, figure 3 B). Like in the whole study population, serum adiponectin concentrations increased in subjects carrying the A allele.

## Discussion

In the present study, we found novel associations between *ADIPOQ *variations and body weight, the risk of T2DM and serum adiponectin concentrations. The results also support the existing view that genetic variation in the *ADIPOQ *influences metabolic and anthropometric characteristics related to T2DM.

### Body weight - SNPs in haploblocks 1 and 2

Rs266729 and rs1501299 are the most extensively studied of the SNPs associating with body weight and BMI in DPS. Earlier studies report associations to various obesity related phenotypes for rs266729 [15,16,29] and for rs1501299 [14,16,17].

We found that rs266729 G allele carriers had higher weight compared with individuals with CC genotype at baseline and a similar trend was seen during the 4-year follow-up, especially in women. Significant association that were seen more often in women than men may be explained by larger number of female subjects in the DPS. In contrast to our results, rs266729 C is a risk allele for obesity related phenotypes in most study populations [15,16]. Genotype differences according to rs1501299 were seen in baseline weight as well as in 4-year follow-up. In agreement with this, the G allele was associated with obesity related phenotypes in some studies [14], but the T allele in others [16,17].

To our knowledge, no previous data concerning associations of intronic SNPs rs16861205 and rs3821799, and rs6773957, located in the 3'UTR, with obesity related phenotypes exists. The strongest association was observed for rs3821799 and it remained significant also in multiple SNP analyses. During the 4-year follow-up, the genotype differences remained constant in both study groups. Thus, individuals with different *ADIPOQ *genotypes did not respond differently to the lifestyle modification regarding weight change.

### Type 2 diabetes - rs266729, rs2241766 and rs2082940

We found significantly increased conversion rates from IGT to T2DM for subjects homozygous for the rs266729 C allele. The G allele, associated with higher body weight, associated with lower T2DM risk in statistical models with or without adjustment for obesity related covariates. It seems therefore that the effect of *ADIPOQ *variation on T2DM risk is independent of its effect on body weight. According to previous studies rs266729 G allele frequently associates with increased risk of T2DM [15,23] and insulin resistance [16,44]. On the other hand, the C allele was associated with increased risk of T2DM in a German study population [25] and with lower clamp-derived insulin sensitivity in a cohort of Europid adolescents and their parents [26].

In addition, the rare minor alleles of rs2241766 and rs2082940 associated with conversion to T2DM in DPS. Rs2241766 is one of the most extensively studied *ADIPOQ *SNPs. Consistent with our results, the G allele associated with T2DM in cross-sectional studies [21], and also with T2DM risk [24] and hyperglycemia [19,20] in prospective studies. On the contrary, others have reported that the T allele associated with elevated insulin/glucose values and insulin resistance [14]. To our knowledge, no previous studies have found association between T2DM and rs2082940, which is in high LD with rs2241766 (r^2 ^= 0.872). It is located in the 3' UTR and its functional role is currently unknown. In multiple SNP analyses rs266729 remained significant predictor of T2DM risk. Thus at least one genetic signal affecting T2DM risk exists in the *ADIPOQ *locus. When the study groups were analysed separately, SNPs associated with T2DM similarly in both study groups. Thus, the effect of lifestyle intervention on T2DM was not modified by the *ADIPOQ *SNPs studied.

### Serum adiponectin levels - SNPs in intron1, exon 2 and in 3'UTR

Serum adiponectin levels are under strong genetic control [45,46]. A genome-wide scan of plasma adiponectin levels provided evidence that variation in *ADIPOQ *gene, especially rs2241766, rs1501299 and +2019A/- insertion/deletion polymorphism, are responsible for linkage of adiponectin levels to 3q27 in the Old Order Amish [28]. Moreover, a recent genome wide association study found that rs17366568 explained 3.8% of variation in adiponectin levels, while 6.7% of the variation was explained by all SNPs in the *ADIPOQ *region [47]. In another whole genome association analysis rs6773957 and rs3774261 associated strongly with adiponectin levels [34]. Furthermore, rs6773957 and two promoter SNPs (rs17300539 and rs822387) associated with higher serum adiponectin levels and a trend for rs17366568 was also seen [33].

In our study, rs16861210, rs17366568 and rs6773957 associated strongly with baseline serum adiponectin levels in a dose dependent manner. In multiple SNP analyses the significance of rs6773957 was lost indicating LD with another, most likely rs17366568. To our knowledge, no previous studies examining the association between rs16861210 and adiponectin levels have been published. Both rs16861210 and rs17366568 are located in intron 1, but are not in strong LD with each other and represent likely independent genetic signals affecting serum adiponectin levels. Functional role of either SNP is currently unknown, but interestingly, three SNPs located immediately on either side of rs17366568 were predicted to affect transcription factor binding sites [47].

The association between rs2241766 and rs2082940 with baseline serum adiponectin remained borderline significant in multi SNP analyses and may represent genetic signal, independent of the variations in intron 1. The association of the rare minor alleles of these two SNPs with higher adiponectin levels in men, and on the other hand, higher risk of T2DM in the total population is contradictory. Especially so, since a non-significant trend for lower adiponectin levels predicting higher risk of developing T2DM was seen in our population. The number of minor allele carriers was relatively low and the results should therefore be interpreted with caution. Nevertheless, the T allele of rs2241766 associates with lower adiponectin levels [14,28,30,31] and the G allele with T2DM related traits in several previous studies [19-21,24].

While association between *ADIPOQ *promoter SNPs and adiponectin levels have been suggested widely [18,23,29,31-33], we did not find significant association between rs266729 and adiponectin levels possibly due to lack of statistical power. The results of several functional studies suggest that rs266729 does not have influence on the transcription efficiency [16,27,32]. On the other hand, an important role in *ADIPOQ *promoter activity was recently suggested for rs266729 and two other promoter SNPs (rs16861194 and rs17300539) [48]. In addition, another study presents evidence that rs266729 alters the sequence in one of the transcriptional stimulatory protein (SP1) binding sites in the promoter region [49]. Unfortunately, in the DPS population, we did not genotype the promoter variant rs17300539 for which important functional role has been also suggested [16,48].

Significant differences were seen in 4-year change in serum adiponectin levels according to rs16861205. Increase in adiponectin levels was greatest in A allele carriers and the most beneficial changes in serum adiponectin concentrations were seen in A allele carriers who were able to lose weight, while in a group of subjects whose weight increased, genotype differences were not seen. This may implicate that A allele carriers benefit more of weight loss in terms of change in adiponectin concentrations. Genotype differences were still significant for the dominant inheritance model when 4-year weight change was used, although the number of subjects was low.

Several *ADIPOQ *association studies have failed to replicate results of previous studies or have even reported opposite effects of alleles. Possible explanation is that the LD patterns differ between populations with various ethnic backgrounds. Moreover, the true functional SNPs underlying complex traits may be rare and population specific. Recently, Bowden at al. (2010) demonstrated that a rare *ADIPOQ *variant explained approximately 17% of the variance in plasma adiponectin in Hispanic American population. Interestingly, this variant was not observed in African American or European American populations [50].

Since *ADIPOQ *alleles have shown opposite effects even in populations with similar ethnic background and disease status [14,51] other explanations, such as differences in participant inclusion criteria, diagnostic criteria and study design, are also possible. As an example, genetic effects for complex traits can vary by age and such interactions can even prevent replication of an association especially in cross-sectional studies [52]. Lastly gene-gene interactions, genetic epistasis and even epigenetic modifications may modify genetic associations. Although these phenomena are still poorly understood in human, Greene et al (2009) have shown that differences in allele frequencies and gene-gene interactions can explained results where effects of gene variations are significant, but in different directions [53]. Lastly, one obvious possibility is that these findings are simply due to chance. However, since significant associations have been replicated in many different populations, the latter alternative seems unlikely.

The two-block LD structure of *ADIPOQ *with the existence of two independent genetic signals corresponding to the haploblock structure reported earlier [22,31] was also found in the DPS and different phenotypes were accounted for mostly by SNPs located in separate regions based on the LD structure of *ADIPOQ*. *ADIPOQ *is a locus of low LD and high haplotypic diversity [54] and it is therefore important to perform the association studies in genetically homogeneous population with comparatively high degree of LD. Owing to its demographic history, the Finnish population exhibits a decrease of genetic diversity and an increase in LD when compared with more admixed populations of central European background and it has been speculated that identifying genes for complex traits could be especially advantageous in this population [55]. Moreover, the participants of the DPS study were carefully selected and phenotyped, hence minimising the risk of false results due to population stratification. Moreover, the follow-up data available in DPS increases the power to find true associations compared to studies with just single measurement of a given variable. The size of the DPS population, however, is moderate for genetic association study and we were only able to measure the serum adiponectin levels from part of the study participants. This may weaken the power to find true associations or increase the risk of false positive findings.

Performing multiple statistical tests in genetic association studies is likely to lead to false positive findings and general guidelines on applying correction for multiple comparisons or threshold values do not exist. In this study, we applied FDR to control for the multiple hypothesis testing and present combination of p-value and q-value for each test performed on single SNPs. The FDR provides an estimation of the minimum false discovery rate at which the test may be called significant. FDR was low (q < 0.1) for associations between *ADIPOQ *SNPs and body weight/BMI and serum adiponectin levels. However, although p value was <0.05, the FDR was higher for the risk of T2DM in our analyses. Nevertheless, given that our candidate gene was selected based on solid prior information on the important role of adiponectin as a metabolic regulator, and the study design that enabled us to perform statistical analyses also on longitudinal data, we believe that our results are true associations. Again, this is further supported by the results of earlier genetic association studies on *ADIPOQ*.

## Conclusions

This study, performed in a homogeneous group of individuals, supported the significant role of *ADIPOQ *variations in conditions linked to T2DM and obesity, partly by replicating earlier findings, but also by discovering novel associations. New evidence for the involvement of rs3821799 and rs6773957 in obesity was provided. Particularly, the strong association of rs6773957 with body weight and serum adiponectin levels is interesting finding as this SNP is located in the 3'UTR and could therefore have an effect on mRNA stability or translational efficiency.

## Competing interests

The authors declare that they have no competing interests.

## Authors' contributions

NS participated in designing the genetic studies, performed the statistical analyses, most of the genotyping and drafted the manuscript. LP participated in designing the genetic studies and writing the manuscript. MK participated in interpretation of data and writing the manuscript. JL, JGE, MV, PIP and SKK contributed to study design and coordination and revised the manuscript. JT and MU are the principle investigators of the DPS study and participated in writing the manuscript. All authors read and approved the final manuscript.

## Pre-publication history

The pre-publication history for this paper can be accessed here:

http://www.biomedcentral.com/1471-2350/12/5/prepub
